# Development of a high-resolution NGS-based HLA-typing and analysis pipeline

**DOI:** 10.1093/nar/gkv184

**Published:** 2015-03-09

**Authors:** Michael Wittig, Jarl A. Anmarkrud, Jan C. Kässens, Simon Koch, Michael Forster, Eva Ellinghaus, Johannes R. Hov, Sascha Sauer, Manfred Schimmler, Malte Ziemann, Siegfried Görg, Frank Jacob, Tom H. Karlsen, Andre Franke

**Affiliations:** 1 Christian-Albrechts-University of Kiel, Institute of Clinical Molecular Biology, Kiel, Germany; 2 Norwegian PSC Research Center, Department of Transplantation Medicine, Division of Cancer Medicine, Surgery and Transplantation, Oslo University Hospital, Rikshospitalet, Oslo, Norway; 3 K.G. Jebsen Inflammation Research Center, Institute of Clinical Medicine, University of Oslo, Oslo, Norway; 4 Research Institute of Internal Medicine, Division of Cancer Medicine, Surgery and Transplantation, Oslo University Hospital, Oslo, Norway; 5 Christian-Albrechts-University of Kiel, Department of Computer Science, Kiel, Germany; 6 Muthesius Academy of Fine Arts and Design, Kiel, Germany; 7 Section of Gastroenterology, Department of Transplantation Medicine, Division of Cancer Medicine, Surgery and Transplantation, Oslo University Hospital, Rikshospitalet, Oslo, Norway; 8 Max-Planck Institute for Molecular Genetics, Berlin, Germany; 9 University of Lübeck, Institute of Transfusion Medicine, Lübeck, Germany

## Abstract

The human leukocyte antigen (HLA) complex contains the most polymorphic genes in the human genome. The classical HLA class I and II genes define the specificity of adaptive immune responses. Genetic variation at the HLA genes is associated with susceptibility to autoimmune and infectious diseases and plays a major role in transplantation medicine and immunology. Currently, the HLA genes are characterized using Sanger- or next-generation sequencing (NGS) of a limited amplicon repertoire or labeled oligonucleotides for allele-specific sequences. High-quality NGS-based methods are in proprietary use and not publicly available. Here, we introduce the first highly automated open-kit/open-source HLA-typing method for NGS. The method employs in-solution targeted capturing of the classical class I (*HLA-A, HLA-B, HLA-C*) and class II HLA genes (*HLA-DRB1, HLA-DQA1, HLA-DQB1, HLA-DPA1, HLA-DPB1*). The calling algorithm allows for highly confident allele-calling to three-field resolution (cDNA nucleotide variants). The method was validated on 357 commercially available DNA samples with known HLA alleles obtained by classical typing. Our results showed on average an accurate allele call rate of 0.99 in a fully automated manner, identifying also errors in the reference data. Finally, our method provides the flexibility to add further enrichment target regions.

## INTRODUCTION

The human leukocyte antigen (HLA) complex is located on chromosome 6p21 and comprises dozens of genes important for immune function (1,2). Of key importance for determining the antigenic specificities of the adaptive immune response, one gene family encoded in the HLA complex comprises the ‘classical’ HLA genes (3,4). Accurate assignment of individual HLA alleles at these loci is essential within several disciplines, e.g. clinical transplantation medicine (5,6), inflammatory disease susceptibility research (7–9), tumor immunology (10–12) and evolutionary biology (13).

Up to recently, HLA typing has mainly been carried out using sequence-specific oligonucleotide probes (SSOP), sequence-specific primers (SSP) and sequence-based typing (SBT) using Sanger sequencing of exons 2 and 3 in class I HLA genes (*HLA-A/B/C*) or exon 2 in class II HLA genes (*HLA-DR/DQ/DP*). High-resolution (i.e. covering all coding variation and by nomenclature consensus at least the first and second field, classically ‘four digits’) typing by means of SSOP and/or SSP usually is an iterative approach that starts with low-resolution typing (i.e. first field, classically two digits), followed by additional characterizations to the extent needed by the application. This process is time consuming and incompatible with any high-throughput research context. Sanger sequencing-based typing has the capability to perform high-resolution typing, but demands polymerase chain reaction (PCR) amplification of individual exons at each locus and often several separate sequencing reactions for each amplicon. Moreover, the results usually contain a large number of cis/trans ambiguities, i.e. heterozygous positions cannot be adequately phased and all alleles matching the sequencing outcome are thus listed as possible genotype combinations. A PCR and Sanger sequencing based method that generates unambiguous HLA typing for four HLA loci was published by Voorter *et al*. (14). Even though it is automatable and delivers reliable results, practical challenges of all PCR-based approaches remain.

In recent years alternative SBT methods using next-generation sequencing (NGS) technology have emerged (15–20). Most of these NGS methods rely on traditional PCR of the target regions followed by massive parallel sequencing of the amplicons. The advantage of NGS is the single strand sequencing nature combined with the increased amount of sequencing reads per sample and locus. This allows for a highly confident allele-determination, hereafter referred to as calling. Because of the single-strand derived NGS reads, the new typing approaches often allow for intragenic phasing between polymorphic nucleotides. However, the limitations of the initial PCR remain for these NGS approaches. Amplicon-based methods are laborious, require extensive PCR primer optimization steps and often demand a manual curation of results.

For targeted NGS, array-based (21) and bead-based (22) enrichment techniques are well established and widely used. The advantages of these oligonucleotide-based enrichments are their ease of use as no extra instrumentation and PCR optimizations are necessary, plus the flexibility to enrich genomic targets of different sizes and complexity. Today whole-exome enrichments are widely used by NGS platforms and different groups worldwide. Major *et al*. (23) published an HLA *in silico* typing approach where whole exome and whole genome NGS data from the 1000 genomes project was employed (24). As exemplified by this application, traditional exome enrichment for HLA genotyping may however result in allelic dropout, because the target baits are designed based on the standard human reference genome sequence not accounting for the allelic variation at the HLA loci. The complexity of the classical HLA loci challenges the development of specific diagnostic-grade enrichment kits. Nevertheless, the collection of known HLA allele sequences is large, captures probably all common alleles and is publicly available (25). Here we present an in-solution targeted enrichment approach for NGS-based HLA genotyping without PCR-based amplification. Our approach consists of a complete turnaround for HLA sequencing including a user-friendly software tool for assigning HLA alleles.

## MATERIALS AND METHODS

### Selection of DNAs for benchmark study

Sample selection was made computationally with the aim of maximizing allelic diversity of the reference set. We started with a collection of 892 cell lines, 584 from the International Histocompatibility Working Group (IHWG) and 308 from the EBRCC-Cell Culture Laboratory in Hannover, Germany. For the complete sample set we ran 100,000 random selections. For each of these selections we performed the following process: one random sample was drawn without putting it back. If a drawn sample had at least one allele that was not drawn before, we put it into the pool of reference samples. Otherwise it was discarded. New samples were drawn until all available reference alleles were in the reference pool. After 100,000 of these runs the pool with the smallest number of samples (*n* = 333) was selected (Supplementary Table S1). During the ordering process it turned out that 36 samples could not be provided. Therefore, additional samples (*n* = 60) from IHWG and the EBRCC-Cell Culture Laboratory in Hannover were manually selected to add diversity to the reduced reference set. The final sample set including all NGS HLA-typing results is listed in Supplementary Table S2.

### Design of RNA baits for targeted enrichment

Commercially available targeted enrichment design normally allows the user to define the genes and genomic targets of interest. The capture probes (baits) are then designed based on reference sequences. In pilot experiments (data not shown) we tested such bait designs targeting only the HLA alleles of the human genome reference sequence. Results from this pilot showed poor concordance between NGS-based and classically determined HLA alleles. This is likely due to failed enrichment because of incomplete reference data when designing the bait set. As expected, the HLA allele enrichment success rate was 100% (data not shown) when DNA from the homozygous HLA reference cell lines were sequenced (26). We hypothesized—although the RNA baits allow for a few mismatches in the hybridization reaction (27)—that an improved bait design was needed to capture the diverse and large number of known HLA alleles. Consequently, we designed an HLA bait panel based on the complete collection of available IMGT/HLA reference sequences. The resulting targeted enrichment showed completeness for our reference panel, at the expense of a loss of evenness.

First a non-overlapping tiling of the hg19 MHC reference sequence was performed to design the RNA baits. This tiling included the chr6 MHC (haplotype *pgf*) plus additional sequences of the available haploid MHC sequences of hg19, i.e. *apd*, *cox*, *dbb*, *mann_mou*, *mcf*, *qbl* and *ssto* (26). Next, all available cDNA and gDNA sequences were downloaded from the IMGT/HLA database version 3.09 (28). In an iterative approach we started aligning all the designed baits against the first gDNA (five mismatches allowed). If an uncovered region with length greater or equal the bait length could be identified, this sequence was tiled to retrieve additional baits. For each gDNA sequence the aforementioned step was repeated. After this process was finished the same procedure was performed on the cDNA collection. For the cDNA collection each exon was treated like a stand-alone sequence. The complete bait design can be found in the Supplementary Data.

### Targeted enrichment, library preparation and sequencing

Targeted enrichment and sample preparation was done with the Agilent^®^ SureSelectXT (http://www.chem.agilent.com) automation kits on a Bravo^®^ (http://www.chem.agilent.com) liquid handling system. The SureSelectXT kit provides library preparation and targeted enrichment in one kit. The amount of input genomic DNA used in our study was 3 μg but more recent protocols like the transposase-based SureSelectQXT (Agilent) require 50 ng only. At our online resource (see QXT folder of the sftp repository; http://www.ikmb.uni-kiel.de/resources/download-tools/software/hlassign) we provide example data for eight samples processed with the SureSelectQXT protocol. The RNA bait length was 120 bp. Sequencing was performed on an Illumina HiSeq2000^®^ (http://systems.illumina.com) with 100 bp paired-end runs. For the targeted enrichment we chose a fragment size between 150 and 300 base pairs and pooled 48 samples per lane. The 48 sample pools provided on average 5,044,060 paired reads per sample (median 4,481,579). We observed an increased error rate for the samples with less read count. So the calling becomes more confident with the higher number of reads obtained. It is difficult to determine an exact lower threshold for high confidence, but taking Supplementary Figure S1 into account we would suggest at least 7 million reads (3.5 million paired reads) as a lower threshold.

### Bioinformatic data analysis

All fastq reads of a sample were aligned against the cDNA collection of the IMGT/HLA database. Importantly, we allowed perfect matches only. The only exception that allowed for truncated alignments were alignments that start and/or end at exon boundaries. Before calling started we reduced the complexity of the data set by reducing the number of mappings to single start point mappings. All cDNA alignments that were not completely covered were discarded. In analogy to Wang *et al*. (20), we also calculated the central read coverage for all cDNAs. If the relation of (central reads)/(noncentral reads) dropped below 0.2 the cDNA was discarded. Of the remaining sequences, all possible allele combinations were analyzed. For both alleles of each allele combination we calculated the coverage as area under the curve (auc), the number of reads that map exclusive to one of the both alleles (allele-specific mappings, asm), the number of mapped pairs per read (mppr) and the length of the mappable sequence (msl). The read equality (req) was calculated as min(asm)/max(asm). After these values were calculated for all allele combinations we scaled the values between 0 and 1 [0;1.0]. The most likely genotype was the one that had the highest harmonic mean for all these measures. Details about the parameter selection and weighting are summarized in the Supplementary Methods. The calling was performed at a Linux cluster (CentOS 5.8, Linux version 2.6.18–308.13.1.el5) with one core per sample and locus.

Weighted harmonic mean

\begin{equation*} H = \frac{{\left( {\sum\nolimits_{k = 1}^5 {\frac{1}{{W_k }}} } \right)*\prod\nolimits_{k = 1}^5 {P_k } }}{{\left( {\sum\nolimits_{k = 1}^5 {\frac{1}{{W_k }}P_k } } \right)}} \end{equation*}

P: The parameters describing the read mapping. These parameters are described in detail above. w: The weighting of the different parameters. P = {asm,req,msl,mppr,auc}; w = {0.5, 1, 0.1, 0.1, 1}

For these parameters the concrete formula is:

\begin{equation*} H = \frac{{(2 + 1 + 10 + 10 + 1)*asm*req * msl*mppr*auc}}{{2*asm + 1*req + 10*msl + 10*mppr + 1*auc}} \end{equation*}

**Figure 1.**
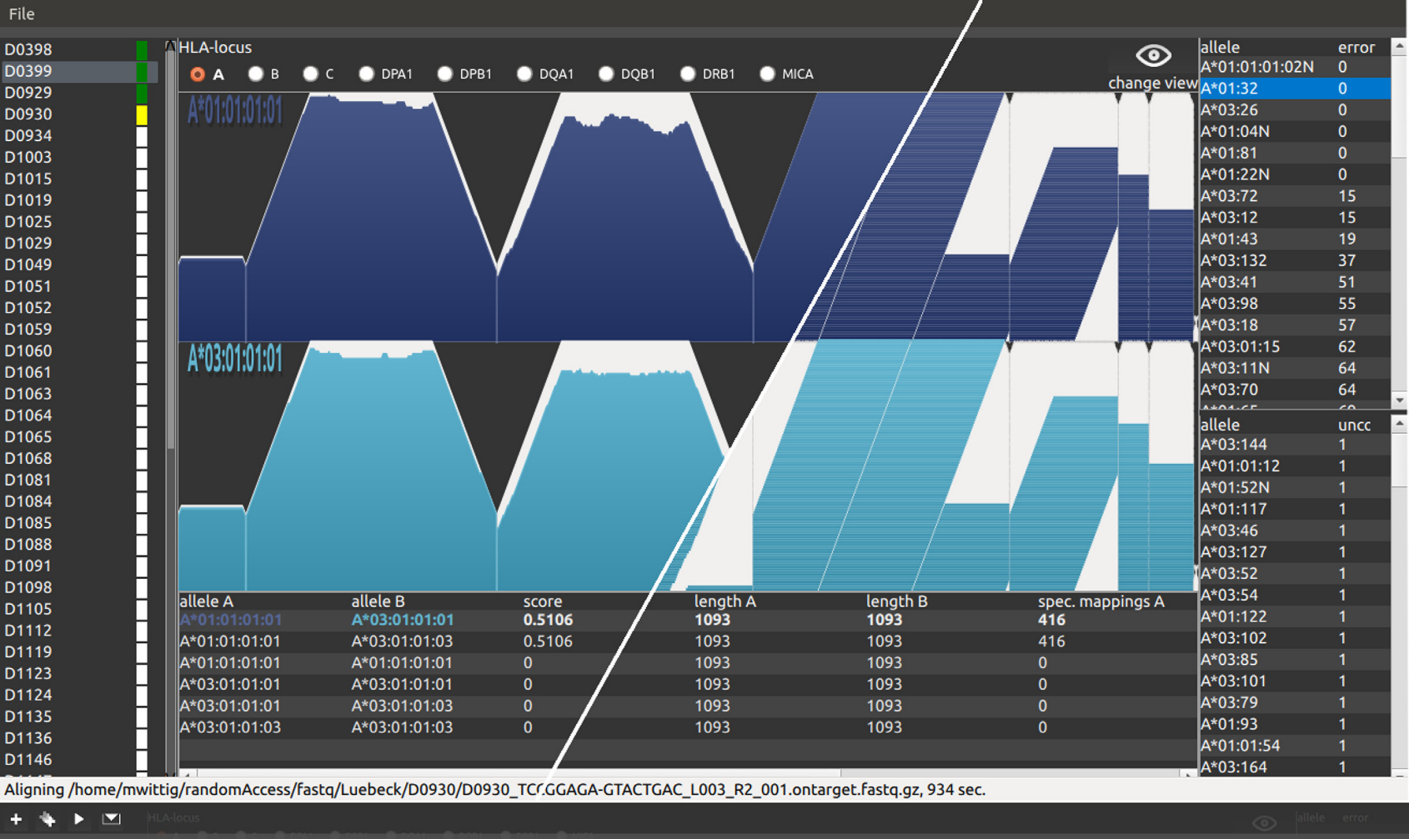
Analysis software. The figure shows a screenshot of the GUI (graphical user interface) of the analysis software. For this figure, we combined two screenshots of two different data views, separated by the white diagonal line. The left part of the graph shows the achieved single start point coverage (see also Figure 2 for further details). The right part shows the corresponding single start point mappings of the same alignment. The different parts of the user interface are as follows: The list view on the left shows samples that are added for analysis. This sample list consists of the sample name followed by a colored rectangle. The colors are coding for the sample states, which are white (added for analysis), yellow (analysis running), green (analysis finished) and red (analysis failed). The middle part is divided into three sections. At the top, the user can select the locus for the selected sample. The ‘change view’ button at the right switches between coverage and read view. Below, the user finds the NGS data visualization of the determined HLA type. The table at the bottom shows a sorted list of the different possible HLA types. The top most HLA type is the most likely determined by the algorithm. The user can change that order to manually correct possible errors. At the right side of the GUI the user finds two additional tables. The upper table contains a sorted list of alleles that failed the initial QC (see Materials and Methods). The column ‘error’ shows the number of nucleotide positions, for which a QC failed value was calculated. The lower table shows the top 50 alleles that were not covered 100%. The table is sorted by the number of uncovered bases in ascending order. Alleles from these two tables can manually be included in the genotype calling. On the other hand, the user can move alleles from the allele calling to one of these tables. This allows for manual evaluation of the calling algorithm. Low covered alleles that failed the initial pre-filtering step can be added. Also degraded DNA, that is not 100% covered, can be analyzed. At the top of the application window, the user finds a small gray bar. When the mouse pointer hovers this area, a toolbox with buttons for sample adding, starting analysis and analysis report moves down (shown at the bottom, below the status bar). A tutorial that contains more details and an example workflow can be found at our online resource http://www.ikmb.uni-kiel.de/resources/download-tools/software/hlassign.

The data analysis that we performed consists of two main steps. The first step is a filtering step, which reduces the number of possible alleles. In a second step the method tries to determine the most likely genotype of the sample. To pass filtering, an allele must have full coverage and in analogy to Wang *et al*. (20) a specific central read coverage. This pre-filtering performs best when the sample has high unique start point coverage. For low coverage samples, the pre-filtering step should be less stringent. So if a sample with low coverage (auc < 0.5) is observed, it is recommended to manually verify the alleles that are filtered out but have a low error (upper right table of Figure 1). The aforedescribed scenario is the main reason for an erroneous result of the fully automated calling. By manual verifying the results in the way described above, it is possible to assign the correct allele in all instances. In the Supplementary Methods we describe how we estimated and verified the parameters we chose for the genotype calling algorithm. The harmonic mean function that we use weighs the parameters differentially. For the parameters asm (weighted half), number of mppr (weighted 0.1) and length of mappable sequence (weighted 0.1), we tested the calling performance for full, half and 0.1 weighted. With the here-in described weighting we achieved the best results for our test set. The test set consisted of 20 samples. For those samples we had classically determined HLA types and HLA types from imputation (29,30). These HLA types had a one-to-two field resolution (Supplementary Methods Table 1). With this data set we were not able to validate our results at a three-field resolution. Nevertheless, we proceeded with that data, determined the above-described weights, for which we achieved an overall genotype concordance of 97% in a fully automated manner, and ordered the validation sample set that we used in this study. With the results of this study, we now present an excellent reference set, representing a broad diversity of HLA alleles. In a future study we will split the larger data set into two parts and use one part for determining the optimal weights and the second part for validation. This optimization will likely increase the accuracy of our automated calling algorithm even further.

**Table 1:** Benchmark statistics for allele calls that could be achieved in a fully automated manner at three-field resolution (note that some of the reference samples only had two-field resolution, Supplementary Table S2)

			Allele call rate		
Locus	Genotypes available	Different alleles	Exact	Including alternatives	Mean alternatives/sample
A	341	114	**0.98**	**0.99**	2.21
B	344	122	**0.99**	**1.00**	1.03
C	285	53	**0.98**	**0.99**	1.58
DRB1	307	80	**0.98**	**0.98**	1.08
DQA1	152	20	**1.00**	**1.00**	0.06
DQB1	264	18	**0.99**	**0.99**	0.06
DPA1	87	5	**0.98**	**0.98**	0.00
DPB1	209	40	**0.97**	**0.97**	0.57

Columns - *Locus*: Lists the typed classical gene locus. *Genotypes available*: Shows the number of samples for which reference genotypes were available. Only alleles for which a reference call was available were used for calculating the call rate. *Different alleles*: Lists the number of different alleles within the reference data set for each locus. The automated calling generates two probable allele calls per diploid sample. *Allele calling exact*: Shows the validated allele call rate that was achieved by the automated calling. *Allele calling including alternatives*: Shows the call rate when including possible alternative alleles (ambiguities). *Mean alternatives/sample*: Shows the average number of alternative allele calls (ambiguities) per sample.

### HLA-typing workflow

Day 1: Perform the DNA library preparation and start the in-solution target capturing protocol until incubation of the hybridization starts. Day 2: Completing the targeted enrichment and pool up to 48 samples. Day 3: Load the sequencing machines and start sequencing. A MiSeq run may be finished after 2 days, a HiSeq run after 10 days. Analysis of the demultiplexed fastq files takes ∼5 min per sample on four cores of an Ubuntu Linux PC with the GUI version.

### Implementation and availability of tool

The analysis software is written in C/C++ code. The graphical version of the software implements multithreading and enables the user to verify the automated HLA calling results (Figure 1). It also has functionality to evaluate and manipulate the calling results and save the results as a comma-separated value (csv) file. It runs at a modern desktop computer but is memory intensive when analyzing many samples in one step. For an analysis of 48 samples we recommend 8GB RAM. Test data and the software can be downloaded at http://www.ikmb.uni-kiel.de/resources/download-tools/software/hlassign. The project described here was performed with a console application on a Linux cluster. The source code and a CentOS executable are provided. The console version utilizes a preinstalled Blat (31) for the alignment and a preinstalled R (32) to generate the graphics. To validate our analysis software we have tested another NGS-based HLA-typing data set that was previously published and that employed random DNA fragmentation. By analyzing this data set with our tool we could confirm the previously reported allele calls (33). Our software handles unpacked and gzipped FASTQ files. If reads are provided in other containers, as in the aforementioned study (33), the user needs to unpack the files before analysis. Moreover, the FASTQ header has to be taken into account. The header type is by default set to Casava 1.8, which is the most recent header format.

## RESULTS

In total, 8159 alleles from the IMGT reference database (28) were considered in our HLA bait panel. The eight loci used for this design were *HLA-A* (*n* = 2019 cDNA sequences), *HLA-B* (*n* = 2600), *HLA-C* (*n* = 1548), *HLA-DRB1* (*n* = 1256), *HLA-DQA1* (*n* = 47), *HLA-DQB1* (*n* = 175), *HLA-DPA1* (*n* = 34) and *HLA-DPB1* (*n* = 155). The final HLA bait panel comprised 16,351 distinct RNA baits, each bait having a length of 120 bp and a cumulative target of 215.5 kb genomic sequence (Supplementary Table S3).

Using the HLA bait panel, we enriched 357 distinct cell line DNAs that were commercially available and for which HLA allele data was previously generated using classical SBT (Supplementary Table S2).

**Figure 2.**
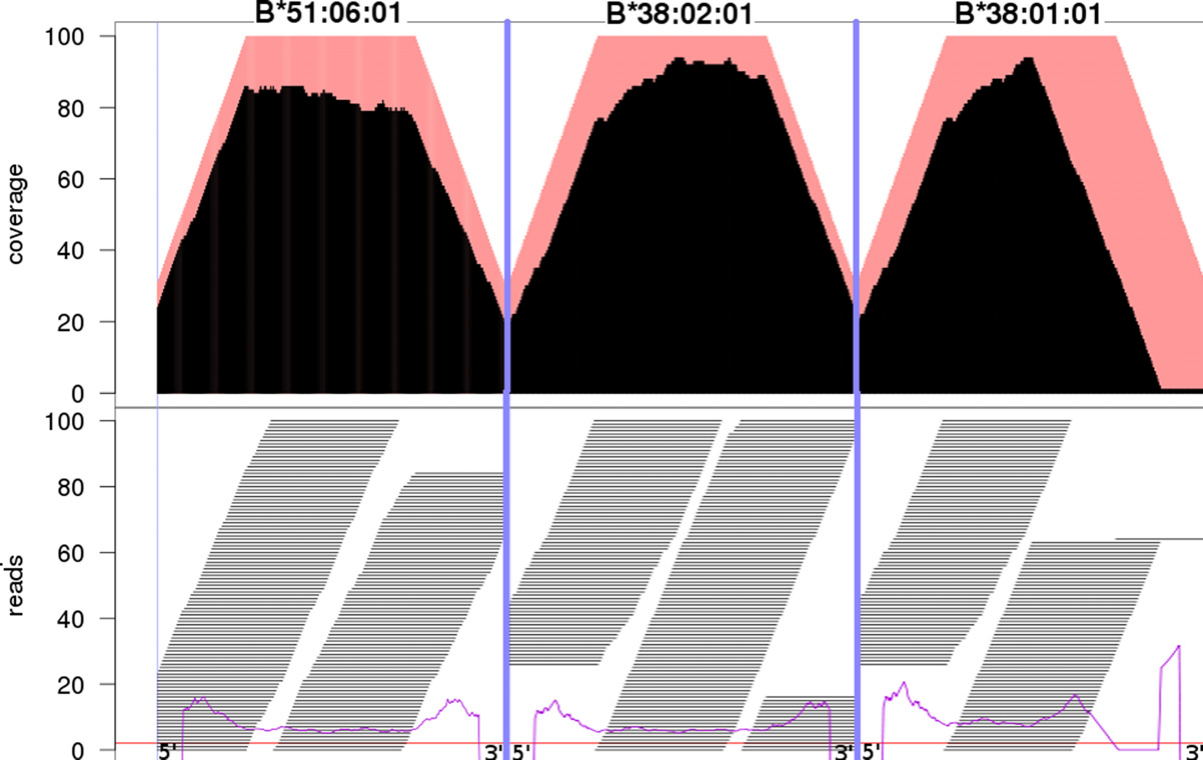
Example of an erroneous reference HLA allele. The picture shows the unique start point coverage (top) and the corresponding short sequencing reads (bottom) of exon 2 for three different *HLA-B* alleles of IHW0994. The red background at the top shows the expected ideal coverage for a perfect single start point mapping with 100 bp reads and a minimum truncated mapping length of 70 bp at the exon boundaries. The black curve shows the sample's unique start point coverage and the corresponding reads are shown in the lower panel of the figure. The reference genotype for *HLA-B* of this sample is 38:01/51:06 as listed by the commercial provider. As shown above, the 3’ part of that exon is not equally covered, thus shows an abnormal read distribution (see purple lines), when considering 38:01 as a candidate allele. As the only difference between 38:01:01 and 38:02:01 are the nucleotides positioned 32 and 34 bases upstream of the 3’ end at exon 2, it is highly likely that the reference data set is wrong. Here we can show that the most probable *HLA-B* genotype of IHW0994 is 38:02/51:06 or 38:02:01/51:06:01 at the 6-digit level. This was later confirmed by Luminex® HLA typing in another laboratory.

Our fully automated calling algorithm achieved a call rate of 0.97–1.00 (Table 1, Materials and Methods). Using visual inspection and manual correction of false calls, the call rate can be further increased. The concordance between the reference and the NGS data set was only 80% for *HLA-DQA1* (Supplementary Table S2), while *HLA-A* showed a concordance of 94% between our results and the available reference. Querying the read mapping suggested that in most cases the discordance was due to errors of the reference data set, i.e. the previous typing methods generated false results (Figure 2, Supplementary Table S4). For example, *HLA-B* of sample IHW09442 has the reference data set genotype 38:01/51:06. Our NGS data in contrast showed that the 3′-prime part of 38:01 at exon 2 could not be identified in the NGS data. However, for the genotype HLA-B*38:02/51:06 we could detect reads covering both cDNAs without gaps (Figure 2). To validate our hypothesis of partially erroneous reference data, 20 of the discordant samples were selected and subjected to Luminex-based HLA typing in an accredited routine diagnostics HLA laboratory. These 20 samples consisted of two batches with 10 samples in each batch. One batch contained samples with presumably erroneous reference data. The second batch comprised samples where we assumed that our method failed to determine the right genotype (Supplementary Table S5). Seventeen of our 20 assumptions were confirmed. For two additional samples, reference errors could be identified, although we assumed it vice versa. For only one sample our assumption was wrong. It turned out that this error was mainly due to issues in the IMGT-reference sequence for DRB1*16:05:01. The reference sequence of DRB1*16:05:01 is incomplete and missing parts had been filled with sequence from 16:01:01, thus the reference allele sequence is a chimeric sequence. Several such potential chimeric sequences were observed in the reference data and, thus excluded from further analysis. In the few other cases (Supplementary Table S2) the discordances were not due to errors in the reference data set but to low coverage, resulting in a homozygous call of a heterozygous sample. A complete list of all calls that were not correctly determined in a fully automated manner is provided through Supplementary Table S6. This table also includes the examples where the correct allele was in the list of possible alternatives.

To our knowledge, most other HLA genotyping methods suggest more than two equally likely genotypes per locus and sample (phase ambiguities). Nevertheless, one allele combination is normally the most likely genotype, as it usually is the most common one in the population under study. However, existing rare alleles are likely alternatives in nearly every case. The mean of possible alternative alleles in our benchmark was 0.75 per locus (median: 1, Supplementary Figure S2).

Using our NGS-based targeted HLA enrichment, genotyping yields highly confident HLA calls in a fully automated manner with only a few ambiguities. We also showed that data visualization and subsequent visual screening allows for manual correction of possible calling errors and further increases accuracy. We provide a graphical user interface for our tool to do the automated calling and manual verification (Figure 1). All analyses can be run on a regular desktop computer under the most common operating systems (Linux, Windows and Mac OS (coming soon)).

## DISCUSSION

In the present study, we have developed the first reliable and open access targeted enrichment approach for high-resolution HLA sequencing, overcoming the limitations of previous commercial enrichment kits. Instead of PCR-based enrichment, we decided to use in-solution capture bait based enrichment that can be performed as an inexpensive single tube reaction. The approach was tested on DNA from 357 cell lines with high accuracy compared to other available HLA genotyping approaches.

The method described here consists of two main steps: (i) targeted in-solution enrichment of the clinically most important HLA loci and (ii) development of a bioinformatic tool for complete data analysis from NGS-derived sequencing reads to HLA types. Recently, other NGS-based HLA -typing approaches were reviewed (18). Most of these approaches rely on NGS of amplicons (20,34). One advantage using amplicon-based approaches is the high specificity and the very effective enrichment. However, the possible size of the amplicon is restricted. In addition, PCR-based targeted enrichment methods often require tedious primer optimization steps to avoid co-amplification of pseudogenes and closely sequence-related HLA loci (e.g. *DRB3*, *DRB4*, *DRB5* versus *DRB1*). Preferential amplification and allelic dropouts (35,36) are further known problems that can occur during the locus-specific PCRs. The advantage of the PCR-free method is the absence of any size limit for the target region. The HLA bait panel thus comprehensively targets the complete HLA genes, including flanking sequences, and may easily be extended by including additional baits for other specific regions like KIR alleles, SNVs for genetic blood group/antigen typing or gender-check variants for Quality Control (QC) purposes, DNA ID tagging Single Nucleotide Variations (SNVs) and even nonhuman targets like Cytomegalovirus known as herpesvirus (CMV) might be added. The HLA panel can also be used as a spike in for other targeted enrichment designs to ensure confident HLA calling in parallel. Furthermore, the straightforward sample handling, which mainly consists of incubation and washing steps, provides a two-day workflow before the sequencing. Therefore, our herein described method does not require obtaining additional instrumentation in most standard sequencing laboratories and is amenable to automation and high-throughput applications. Recently, Major *et al*. (23) published a high-throughput HLA genotyping approach of whole genome data from the 1000 genomes project. Although Major *et al*. analyzed class I loci only, they demonstrated that a low sequencing coverage introduces uncertainties to the results. For the low-depth whole genome data, the success rate was ∼0.85 at the two-field resolution level. With our targeted enrichment the achieved unique start point coverage is greater 50x.

The on-target read mapping rate of our in-solution targeted enrichment is around 5%, when allowing for perfect matches only. Assuming that our target region reflects only around 0.007% of the human genome (215.5 kb out of 3.23 Gb for total genome), the target was enriched 700-fold. With current instruments (Illumina's HiSeq 2000) we are able to pool 48 samples in one lane. Increasing the on-target rate in the future will further increase the possible number of samples that can be analyzed in parallel. We anticipate that the method will also work with other sequencing technologies, especially the MiSeq system from Illumina, which showed equally good results in our laboratory (data not shown, but MiSeq reads are downloadable via our online repository). Employing the MiSeq, the complete turnaround time can be further reduced. However, currently only five samples per MiSeq run are recommended.

Our proposed method is well suited to identify annotated HLA alleles in large sample sets. A current weakness of our analysis approach is that the method is not able to identify novel and not yet annotated alleles in an automated fashion. However, our software tool suggests such potentially novel alleles to the user. A potential hint to a novel allele can be a no call for one gene locus while other loci show clear calls and high coverage, for example. Another scenario is erroneous homozygous calls where the second not identified allele is a new allele. In such a case at least one of the two tables at the right side of the GUI contains alleles that show a high sequence similarity to the new allele. So if a homozygous call is identified and diverse alleles (regarding sequence similarity) are listed at the tables on the right side, the presence of a new allele is likely. In such instances another independent HLA typing or analysis method of the same sample is recommended. For this, we would suggest a conservative mapping and phasing (33) or a *de novo* assembly approach (37) for which the already obtained data can be used. Another recommended approach would be a full-length sequencing approach (14) if available for the given locus and if an additional DNA sample preparation is acceptable. Given the ability to detect these instances, and the comprehensiveness of current allele databases, we consider the advantages of the approach still too far outweigh these rare occasions of additional typing efforts.

In summary, we have developed an open-source enrichment kit and comprehensive bioinformatic package for accurate, high-throughput, high-resolution HLA typing, effectively by-passing the laborious work step of PCR-based enrichment prior to NGS. To allow scientists to verify their results, we provide a user-friendly graphical user interface. The current version of the software can be downloaded at http://www.ikmb.uni-kiel.de/resources/download-tools/software/hlassign.

## SUPPLEMENTARY DATA

Supplementary Data are available at NAR Online.

SUPPLEMENTARY DATA
