# Supplementary material for: Development of a high-resolution NGS-based HLA-typing and analysis pipeline
Source: Nucleic Acids Res. 2015 Mar 9;43(11):e70. doi: 10.1093/nar/gkv184 (PMC4477639; doi:10.1093/nar/gkv184)
Supplement: SUPPLEMENTARY DATA [file supp_43_11_e70__index.html]

Development of a high-resolution NGS-based HLA-typing and analysis pipeline — Development of a high-resolution NGS-based HLA-typing and analysis pipeline — SUPPLEMENTARY DATA 

# Development of a high-resolution NGS-based HLA-typing and analysis pipeline

## SUPPLEMENTARY DATA

**Files in this Data Supplement:**

- SUPPLEMENTARY DATA
- SUPPLEMENTARY DATA
- SUPPLEMENTARY DATA
- SUPPLEMENTARY DATA
