# Supplementary material for: Development of a high-resolution NGS-based HLA-typing and analysis pipeline
Source: Nucleic Acids Res. 2015 Mar 9;43(11):e70. doi: 10.1093/nar/gkv184 (PMC4477639; doi:10.1093/nar/gkv184)
Supplement: SUPPLEMENTARY DATA [file supp_gkv184_nar-02838-met-k-2014-File004.pdf]

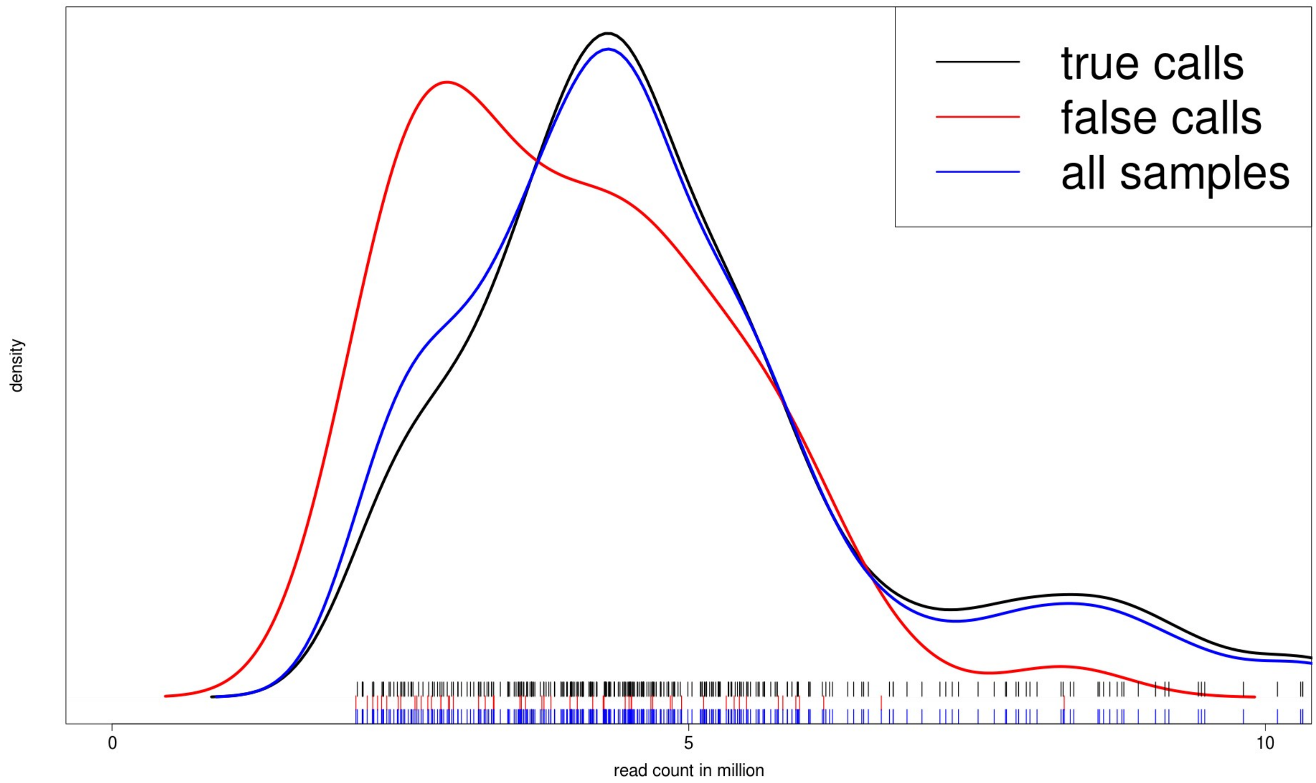


**Supplementary Figure S1: Read-depth vs. error rate**
Read counts per sample with respect to HLA calling accuracy. The above picture shows three density curves and three rows with bars at the bottom. Each bar represents the read count of a sample and the number of reads per sample can be taken from the X axis. The curves above are the corresponding density curves for these data. There are three groups with different colors. Blue is for the complete sample set. Black for samples with correct allele calls for all loci and red for samples with at least one false call. While the blue and black curves are very similar, one can see the higher error rate for samples with lower read count. The graph is limited to 10 million at the X axis, but there are no false calls samples above this cut off.

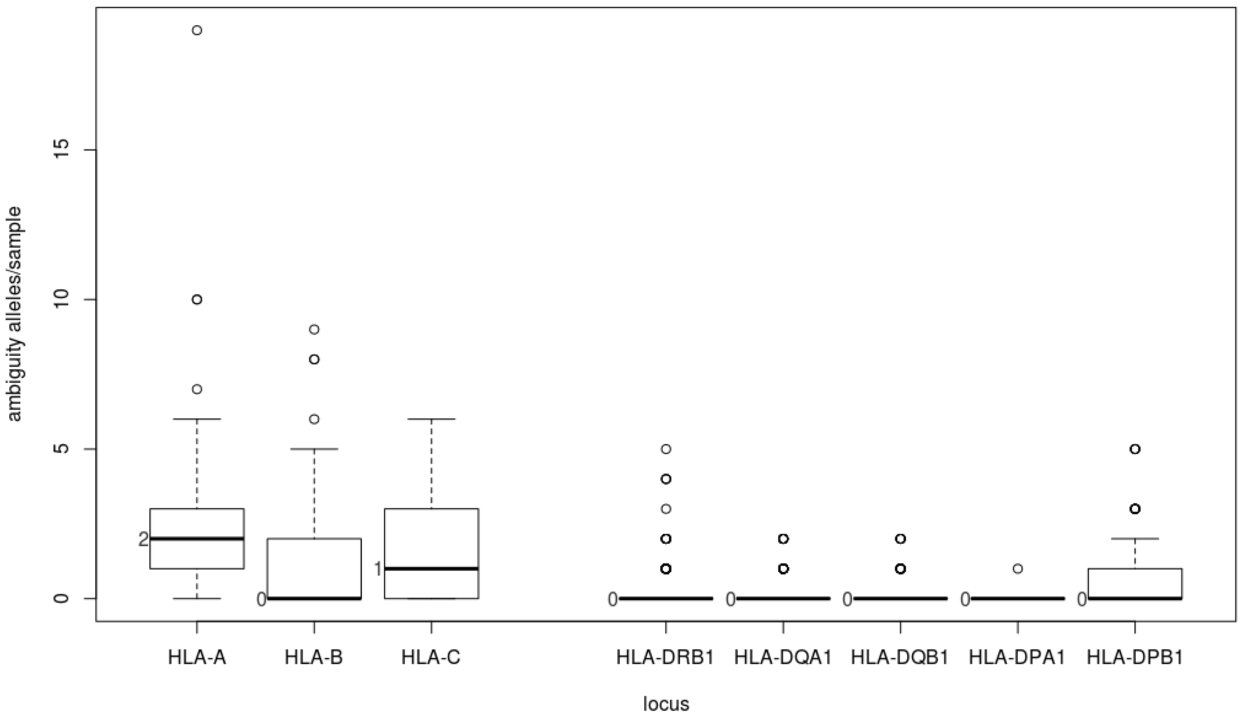


**Supplementary Figure S2: Alternative alleles per locus**
The eight boxplots above show the distribution of the number of possible alternative alleles for each of the analyzed loci. For class II loci the median is always zero. For class I we found more alternatives per sample and locus, but the 75 percent quantile is only three for *HLA-A* and *HLA-C*, and two for *HLA-B*.
