# Supplementary material for: Development of a high-resolution NGS-based HLA-typing and analysis pipeline
Source: Nucleic Acids Res. 2015 Mar 9;43(11):e70. doi: 10.1093/nar/gkv184 (PMC4477639; doi:10.1093/nar/gkv184)
Supplement: SUPPLEMENTARY DATA [file supp_gkv184_nar-02838-met-k-2014-File006.pdf]

SUPPLEMENTARY METHODS

Description of parameter selection for the automated calling algorithm

The first analyses of the HLA data were performed with the haploid cell lines described by Horton *et al.* (1). For each sample and locus we mapped all reads to the collection of available reference sequences and saw that the allele with the highest number of perfect alignable reads was the reference allele. For the evaluation of heterozygous samples we had to generate a valid sample set. Here we chose 20 samples for which we had Sanger-determined HLA allele data available (see **Supplementary Methods Table 1** below). For these samples we performed the targeted enrichment with a bait design that also covered the Affymetrix SNP array 6.0 SNVs of the HLA region. Employing our pibase software (2) for SNP calling and HLA imputation (3), we calculated two field HLA types for that sample set (Supplementary Methods Table 1). These twenty samples and their Sanger and imputation based HLA types were the reference data with which we set up the analysis method.

Next, we ran the afore-mentioned perfect mapping count approach on the heterozygous samples. When we sorted all alleles by the number of mapped reads (descending), we found allele A at the top of the list followed by many alleles that showed high similarity to that allele A. Allele B was very rarely ranked second place. Therefore, additional parameters had to be established to identify the correct allele combination for the sample's genotype. We ended up with 5 parameters:

First parameter

As described in the **Methods** section of the main manuscript we removed all redundancies regarding the mapped reads. Based on these results we calculated an ideal coverage, which reflects the maximal possible coverage. The **area under the curve** (**AUC**) was calculated as the fraction of ideal coverage that was achieved. The AUC parameter was then used instead of the number of perfect alignable reads. In **Supplementary Methods Figure 1a** we visualized the distribution of the AUC for all possible genotypes built from all fully covered alleles. The distribution for the reference genotypes is shown in blue. One can see that the AUC of the reference genotype is usually at the upper end of the distribution.

Second parameter

For heterozygous samples we assumed to cover the SNV positions of both DNA copies with about the same number of reads. Taking this into account we calculated the **read equality** (**REQ**) and counted the read mappings that mapped to only one of both alleles of a genotype. Then we divided the minimum of both by the maximum of both and received a value between 0 and 1 (1 stands for an exact even distribution). **Supplementary Methods Figure 1b** shows that the read equality of the reference genotypes is again close to the upper-side of the distribution. The zero values come from the homozygous genotypes.

Third parameter

A further review of the results showed that not only the read equality is a criteria that is maximized for heterozygous samples, but also the number of **allele specific mappings** per base (**ASM**). The ditribution of ASM is visualized in **Supplementary Methods Figure 2a**. The values of the reference genotypes are again located at the upper side of the destribution, but less than it is for the first two parameters (**Supplementary Methods Figure 1**). This observation already indicates a probable minor weighting of this parameter (compared to auc and req) when we try to find the genotype that has all parameters as high as possible later.

Fourth parameter

For the 4th parameter we calculated the **number of mapped pairs per read** (**MPPR**) for each allele of every genotype. In case the input data comes from a paired end run, we assumed that the correct allele mapping shows as many paired end mappings as possible. The distribution of these values is shown in **Supplementary Methods Figure 2b**. The distribution again indicates a minor weighting of that parameter.

Fifth parameter

It also turned out that the IMGT/HLA database often only has sequence information for a reduced number of exons. These are, as expected, exons 2 & 3 for class I and exon 2 for class II genes. These short cDNA references have the tendency to show high values for allele specific mappings per base. On the other hand these short cDNAs cannot explain where many of the locus specific reads stem from. To correct for such instances, we introduced the **length of the mappable sequence** (**MSL**) as parameter five. For this parameter we assumed that the longer allele version is present if we detect the sequence for this allele. The distribution of these values is shown in **Supplementary Methods Figure 2c** and a minor weighting is again indicated.

After all parameters were calculated for all genotypes, they were scaled between zero and one for each sample and locus separate. If a value was by definition already scaled between zero and one this step was skipped (AUC, REQ). In the next step we calculated the harmonic mean for the rescaled parameters. Like the distribution of the values already indicated, some parameters might be weighted less. For ASM, MPPR and MSL we tested the weightings 1, 0.5 and 0.1. The best results for our validation set (**Supplementary Methods Table 1**) could be achieved with ASM weighted 0.5, MPPR weighted 0.1 and MSL weighted 0.1. The exact formula can be found in the **Methods** section of the main manuscript.

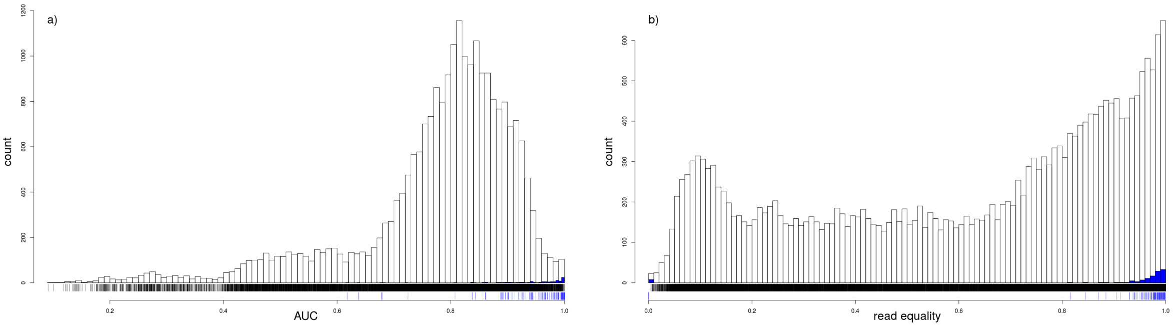

Supplementary Methods Figure 1: Distribution of the parameter values that were used for the automated calling algorithm. a) Histograms of the area-under-the-curve and b) read-equality. For every sample and all loci we took all the alleles that were covered by 100%, following our mapping criteria described in the publication. For each locus we build all possible genotypes of the available alleles and calculated the parameters AUC (area under the curve) and read equality (see main paper). The values were sample- and locus-specific scaled between zero and one ([0;1]). The histograms for these values are shown here. The black framed bars were generated from the whole data set, the blue bars are derived from the values calculated for the reference alleles. We see for both parameters that these values are at the upper end of the distribution. The zero values for read equality stem from the homozygous genotypes. The panel between the x-axes and axis labels show vertical lines for each value that was used to to generate the histogram. Black lines are the values for the black framed histogram and blue for the blue histogram.

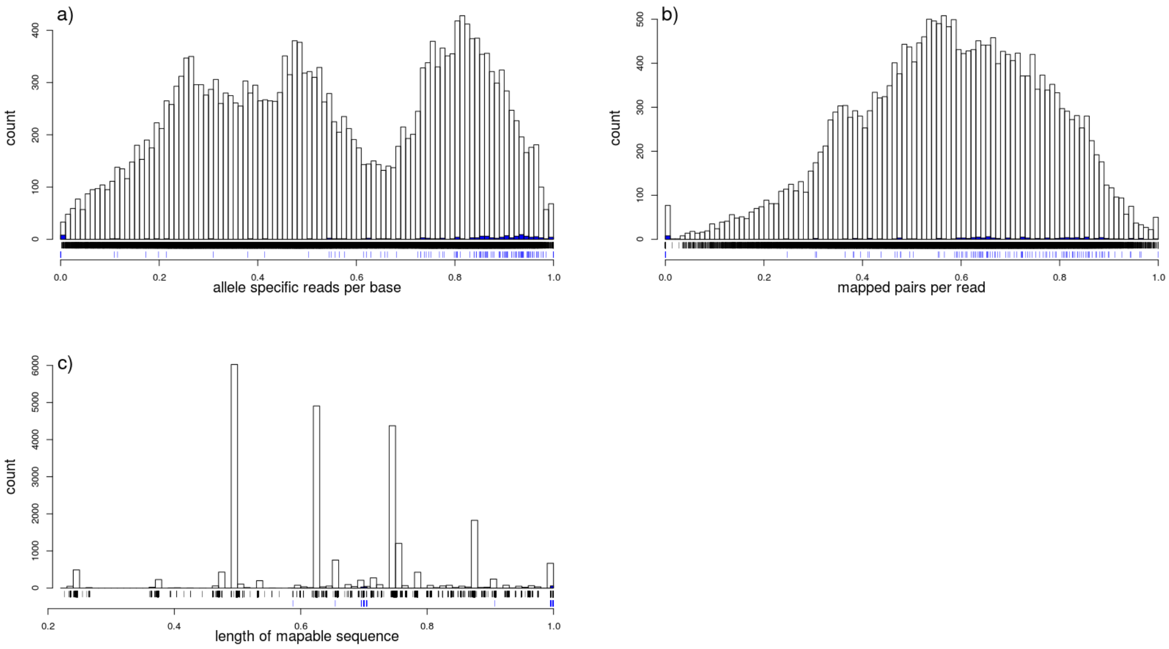

Supplementary Methods Figure 2: Distribution of the parameter values that were used for the automated calling algorithm. Please see legend of Supplementary Methods Figure 1 for a description of the histogram type. The parameters visualized here are a) allele specific reads per base, b) mapped pairs per read and c) length of the mappable sequence. For the allele specific reads per base one can see a tendency that the reference genotypes are concentrated at the upper side of the distribution even in instances where it is not so clear as for the AUC and the read equality. For the score calculation we weigh allele specific reads per base with 0.5. For the remaining two parameters this effect is less strong and we weight those with 0.1 only.

Supplementary Methods Table 1. Reference data set that was used for the implementation of the calling algorithm. The table shows the sample set used to develop the HLA calling algorithm. The first column listst the sample identifier, the second the HLA locus for which the following columns show the allele calls. Columns 3 and 4 show both alleles determined by the *in silico* SNP2HLA allelle imputation. Columns 5 and 6 show the classically Sanger determined genotype. Columns 7 and 8 show the HLA calling results of our tool after manual review of the NGS based calls. The fully automated calling that was based on our feature selection and weighting showed an overall 97% concordance to these results.

		HLA imputation		Sanger		NGS based caling	
Sample	locus	allele 1	allele 2	allele 1	allele 2	allele 1	allele 2
B0708	HLA-A	A*01:01	A*31:01	A*01	A*19	A*01:01:01	A*31:01:02
B0708	HLA-B	B*08:01	B*40:01	B*08	B*40	B*08:01:01	B*40:01:02
B0708	HLA-C	C*03:04	C*07:01	C*03	C*07	C*03:04:01	C*07:01:01
B0708	HLA-DQA	DQA*03:01	DQA*05:01			DQA1*03:01:01	DQA1*05:01:01
B0708	HLA-DQB	DQB*02:01	DQB*03:02	DQB1*02:01	DQB1*03:02	DQB1*02:01:01	DQB1*03:02:01
B0708	HLA-DRB1	DRB1*03:01	DRB1*04:04	DRB1*03:01	DRB1*04:04	DRB1*03:01:01	DRB1*04:04:01
B0709	HLA-A	A*01:01	A*03:01	A*01	A*03:26	A*01:01:01	A*03:26
B0709	HLA-B	B*08:01	B*35:03	B*08	B*35	B*08:01:01	B*35:03:01
B0709	HLA-C	C*04:01	C*07:01	C*04	C*07	C*04:01:01	C*07:01:01
B0709	HLA-DQA	DQA*03:01	DQA*05:01			DQA1*03:01:01	DQA1*05:01:01
B0709	HLA-DQB	DQB*02:01	DQB*03:02	DQB1*02:01	DQB1*03:02	DQB1*02:01:01	DQB1*03:02:01
B0709	HLA-DRB1	DRB1*03:01	DRB1*04:04	DRB1*03:01	DRB1*04:03	DRB1*03:01:01	DRB1*04:03:01
B0710	HLA-A	A*02:01	A*24:02	A*02	A*24:02	A*02:01:01	A*24:02:01
B0710	HLA-B	B*35:01	B*40:01	B*35	B*40	B*35:01:01	B*40:01:02
B0710	HLA-C	C*03:04	C*04:01	C*03	C*04	C*03:04:01	C*04:01:01
B0710	HLA-DQA	DQA*01:01	DQA*03:01			DQA1*01:01:01	DQA1*03:01:01
B0710	HLA-DQB	DQB*03:02	DQB*05:01	DQB1*06:02	DQB1*06:03	DQB1*03:02:01	DQB1*05:01:01
B0710	HLA-DRB1	DRB1*01:01	DRB1*04:04	DRB1*01:03	DRB1*04:04	DRB1*01:03	DRB1*04:04:01
B0711	HLA-A	A*02:01	A*02:01	A*02	A*02	A*02:01:01	A*02:01:01
B0711	HLA-B	B*27:05	B*40:01	B*27	B*40	B*27:05:02	B*40:01:02
B0711	HLA-C	C*02:02	C*03:04	C*02	C*03	C*02:02:02	C*03:04:01
B0711	HLA-DQA	DQA*01:02	DQA*01:03			DQA1*01:02:01	DQA1*01:03:01
B0711	HLA-DQB	DQB*06:03	DQB*06:04	DQB1*06:03	DQB1*06:04	DQB1*06:03:01	DQB1*06:04:01
B0711	HLA-DRB1	DRB1*13:01	DRB1*13:02	DRB1*13:01	DRB1*13:02	DRB1*13:01:01	DRB1*13:02:01
B0712	HLA-A	A*01:01	A*02:01	A*01:01	A*02:01	A*01:01:01	A*02:01:01
B0712	HLA-B	B*15:01	B*39:06	B*14	B*16	B*15:01:01	B*39:06:02
B0712	HLA-C	C*03:03	C*07:02	C*03	C*07	C*03:03:01	C*07:02:01
B0712	HLA-DQA	DQA*01:03	DQA*04:01			DQA1*01:03:01	DQA1*04:01:01
B0712	HLA-DQB	DQB*04:02	DQB*06:03	DQB1*04	DQB1*06:03	DQB1*04:02:01	DQB1*06:03:01
B0712	HLA-DRB1	DRB1*08:01	DRB1*13:01	DRB1*08:01	DRB1*13:01	DRB1*08:01:01	DRB1*13:01:01
B0713	HLA-A	A*01:01	A*68:01	A*01:01	A*68:01	A*01:01:01	A*68:01:02
B0713	HLA-B	B*51:01	B*57:01	B*05	B*17	B*51:01:01	B*57:01:01
B0713	HLA-C	C*06:02	C*14:02	C*06	C*14	C*06:02:01	C*14:02:01
B0713	HLA-DQA	DQA*01:01	DQA*01:03			DQA1*01:01:01	DQA1*01:03:01
B0713	HLA-DQB	DQB*05:01	DQB*06:03	DQB1*05:01	DQB1*06:03	DQB1*05:01:01	DQB1*06:03:01
B0713	HLA-DRB1	DRB1*01:01	DRB1*13:01	DRB1*01:01	DRB1*13:01	DRB1*01:01:01	DRB1*13:01:01

Sample	locus	HLA imputation allele 1	HLA imputation allele 2	Sanger allele 1	Sanger allele 2	NGS based caling allele 1	NGS based caling allele 2
B0714	HLA-A	A*01:01	A*24:02	A*01:01	A*24:02	A*01:01:01	A*24:02:01
B0714	HLA-B	B*08:01	B*51:01	B*05	B*08	B*08:01:01	B*51:01:01
B0714	HLA-C	C*07:01	C*15:02	C*07	C*15	C*07:01:01	C*15:02:01
B0714	HLA-DQA	DQA*01:03	DQA*05:01			DQA1*01:03:01	DQA1*05:01:01
B0714	HLA-DQB	DQB*02:01	DQB*06:03	DQB1*02:01	DQB1*06:03	DQB1*02:01:01	DQB1*06:03:01
B0714	HLA-DRB1	DRB1*03:01	DRB1*13:01	DRB1*03:01	DRB1*13:01	DRB1*03:01:01	DRB1*13:01:01
B0715	HLA-A	A*02:01	A*11:01	A*02:01	A*11:01	A*02:01:01	A*11:01:01
B0715	HLA-B	B*15:01	B*44:02	B*12	B*15	B*15:01:01	B*44:02:01
B0715	HLA-C	C*03:03	C*05:01	C*03	C*05	C*03:03:01	C*05:01:01
B0715	HLA-DQA	DQA*01:02	DQA*01:03			DQA1*01:02:01	DQA1*01:03:01
B0715	HLA-DQB	DQB*06:02	DQB*06:03	DQB1*06:02	DQB1*06:03	DQB1*06:02:01	DQB1*06:03:01
B0715	HLA-DRB1	DRB1*13:01	DRB1*15:01	DRB1*13:01	DRB1*15:01	DRB1*13:01:01	DRB1*15:01:01
B0716	HLA-A	A*31:01	A*68:01	A*19:01	A*28	A*31:01:02	A*68:01:02
B0716	HLA-B	B*27:05	B*35:01	B*27	B*35	B*27:05:02	B*35:01:01
B0716	HLA-C	C*01:02	C*14:02	C*01	C*14	C*01:02:01	C*14:02:01
B0716	HLA-DQA	DQA*01:01	DQA*01:02			DQA1*01:01:01	DQA1*01:02:01
B0716	HLA-DQB	DQB*05:01	DQB*06:02	DQB1*05:01	DQB1*06:02	DQB1*05:01:01	DQB1*06:02:01
B0716	HLA-DRB1	DRB1*01:01	DRB1*15:01	DRB1*01:01	DRB1*15:01	DRB1*01:01:01	DRB1*15:01:01
B0717	HLA-A	A*02:01	A*68:01	A*02	A*28	A*02:01:01	A*68:01:01
B0717	HLA-B	B*13:02	B*35:03	B*13	B*35	B*13:02:01	B*35:03:01
B0717	HLA-C	C*04:01	C*06:02	C*04	C*06	C*04:01:01	C*06:02:01
B0717	HLA-DQA	DQA*01:01	DQA*02:01			DQA1*01:01:01	DQA1*02:01
B0717	HLA-DQB	DQB*02:02	DQB*05:01	DQB1*05:01	DQB1*02:02	DQB1*02:02:01	DQB1*05:01:01
B0717	HLA-DRB1	DRB1*01:01	DRB1*07:01	DRB1*01:01	DRB1*07:01	DRB1*01:01:01	DRB1*07:01:01
B0718	HLA-A	A*24:02	A*29:02	A*09	A*19	A*24:02:01	A*29:02:01
B0718	HLA-B	B*07:02	B*44:03	B*07	B*12	B*07:02:01	B*44:03:01
B0718	HLA-C	C*07:02	C*16:01	C*07	C*16	C*07:02:01	C*16:01:01
B0718	HLA-DQA	DQA*01:01	DQA*02:01			DQA1*01:04:01	DQA1*02:01
B0718	HLA-DQB	DQB*02:02	DQB*05:03	DQB1*05:03	DQB1*02:02	DQB1*02:02:01	DQB1*05:03:01
B0718	HLA-DRB1	DRB1*07:01	DRB1*14:01	DRB1*07:01	DRB1*14:01	DRB1*07:01:01	DRB1*14:54:01
B0719	HLA-A	A*03:01	A*03:01	A*03	A*03	A*03:01:01	A*03:01:01
B0719	HLA-B	B*07:02	B*44:02	B*07	B*12	B*07:02:01	B*44:02:01
B0719	HLA-C	C*05:01	C*07:02	C*05	C*07	C*05:01:01	C*07:02:01
B0719	HLA-DQA	DQA*01:01	DQA*01:02			DQA1*01:02:01	DQA1*05:05:01
B0719	HLA-DQB	DQB*03:01	DQB*06:02	DQB1*03:01	DQB1*06:02	DQB1*03:01:01	DQB1*06:02:01
B0719	HLA-DRB1	DRB1*01:03	DRB1*15:01	DRB1*01:03	DRB1*15:01	DRB1*01:03	DRB1*15:01:01
B0720	HLA-A	A*25:01	A*26:01	A*10	A*10	A*25:01:01	A*26:01:01
B0720	HLA-B	B*08:01	B*15:01	B*08	B*15	B*08:01:01	B*15:01:01
B0720	HLA-C	C*03:03	C*07:01	C*03	C*07	C*03:03:01	C*07:01:01
B0720	HLA-DQA	DQA*03:01	DQA*05:01			DQA1*03:03:01	DQA1*05:01:01
B0720	HLA-DQB	DQB*02:01	DQB*03:01	DQB1*02:01	DQB1*03:01	DQB1*02:01:01	DQB1*03:01:01
B0720	HLA-DRB1	DRB1*03:01	DRB1*04:01	DRB1*03:01	DRB1*04:08	DRB1*03:01:01	DRB1*04:08:01

		HLA imputation		Sanger		NGS based caling	
Sample	locus	allele 1	allele 2	allele 1	allele 2	allele 1	allele 2
B0721	HLA-A	A*01:01	A*02:01	A*01	A*02	A*01:01:01	A*02:01:01
B0721	HLA-B	B*15:01	B*57:01	B*15	B*17	B*15:01:01	B*57:01:01
B0721	HLA-C	C*03:03	C*06:02	C*03	C*06	C*03:03:01	C*06:02:01
B0721	HLA-DQA	DQA*01:03	DQA*01:03			DQA1*01:03:01	DQA1*01:03:01
B0721	HLA-DQB	DQB*06:03	DQB*06:03	DQB1*06:03	DQB1*06:03	DQB1*06:03:01	DQB1*06:03:01
B0721	HLA-DRB1	DRB1*13:01	DRB1*13:01	DRB1*13:01	DRB1*13:02	DRB1*13:01:01	DRB1*13:01:01
B1512	HLA-A	A*01:01	A*02:01	A*01:01:01	A*02:01:01:01	A*01:01:01	A*02:01:01
B1512	HLA-B	B*35:01	B*52:01	B*35:01:01	B*52:01:01	B*35:01:01	B*52:01:01
B1512	HLA-C	C*04:01	C*12:02	C*04:01:01:01	C*12:02:01	C*04:01:01	C*12:02:02
B1512	HLA-DQA	DQA*01:01	DQA*01:01			DQA1*01:01:01	DQA1*01:04:01
B1512	HLA-DQB	DQB*05:01	DQB*05:03	DQB1*05:01	DQB1*05:03	DQB1*05:01:01	DQB1*05:03:01
B1512	HLA-DRB1	DRB1*01:01	DRB1*14:01	DRB1*01:01	DRB1*14:11	DRB1*01:01:01	DRB1*14:11
B1513	HLA-A	A*01:01	A*02:01	A*01:02	A*02:01/09	A*01:02	A*02:01:01
B1513	HLA-B	B*14:01	B*27:05	B*27:05	B*81:01	B*27:05:02	B*81:01
B1513	HLA-C	C*02:02	C*08:02	C*02:02	C*08:04	C*02:02:02	C*08:04:01
B1513	HLA-DQA	DQA*01:01	DQA*03:01			DQA1*01:05	DQA1*03:01:01
B1513	HLA-DQB	DQB*03:02	DQB*05:01	DQB1*01:05	DQB1*03:01	DQB1*03:02:01	DQB1*05:01:01
B1513	HLA-DRB1	DRB1*04:04	DRB1*13:01	DRB1*04:04	DRB1*12:01	DRB1*04:04:01	DRB1*12:01:01
B1985	HLA-A	A*01:01	A*02:01	A*01	A*02	A*01:01:01	A*02:01:01
B1985	HLA-B	B*44:02	B*57:01	B*12	B*17	B*44:02:01	B*57:01:01
B1985	HLA-C	C*05:01	C*06:02	C*05	C*06	C*05:01:01	C*06:02:01
B1985	HLA-DQA	DQA*03:01	DQA*05:05			DQA1*03:03:01	DQA1*05:05:01
B1985	HLA-DQB	DQB*03:01	DQB*03:01	DQB1*03:01	DQB1*03:01	DQB1*03:01:01	DQB1*03:01:01
B1985	HLA-DRB1	DRB1*04:01	DRB1*12:01	DRB1*04:01	DRB1*12:01	DRB1*04:01:01	DRB1*12:01:01
B1986	HLA-A	A*02:01	A*32:01	A*02	A*19	A*02:01:01	A*32:01:01
B1986	HLA-B	B*40:01	B*44:02	B*0	B*40	B*40:01:02	B*40:01:02
B1986	HLA-C	C*03:04	C*05:01	C*0	C*03	C*03:04:01	C*05:01:01
B1986	HLA-DQA	DQA*03:01	DQA*05:05			DQA1*03:01:01	DQA1*03:01:01
B1986	HLA-DQB	DQB*03:02	DQB*03:03	DQB1*03:02	DQB1*03:03	DQB1*03:02:01	DQB1*03:03:02
B1986	HLA-DRB1	DRB1*04:01	DRB1*09:01	DRB1*04:01	DRB1*09:01	DRB1*04:01:01	DRB1*09:01:02
B1987	HLA-A	A*01:01	A*03:01	A*01	A*03	A*01:01:01	A*03:01:01
B1987	HLA-B	B*08:01	B*15:01	B*08	B*15	B*08:01:01	B*15:01:01
B1987	HLA-C	C*03:04	C*07:01	C*03	C*07	C*03:04:01	C*07:01:01
B1987	HLA-DQA	DQA*01:01	DQA*05:01			DQA1*01:05	DQA1*05:01:01
B1987	HLA-DQB	DQB*02:01	DQB*05:01	DQB1*02:01	DQB1*05:01	DQB1*02:01:01	DQB1*05:01:01
B1987	HLA-DRB1	DRB1*03:01	DRB1*10:01	DRB1*03:01	DRB1*10:01	DRB1*03:01:01	DRB1*10:01:01
B1988	HLA-A	A*01:01	A*03:01	A*01	A*03	A*01:01:01	A*03:01:01
B1988	HLA-B	B*37:01	B*44:02	B*12	B*37	B*37:01:01	B*44:27:01
B1988	HLA-C	C*06:02	C*07:04	C*06	C*07	C*06:02:01	C*07:04:01
B1988	HLA-DQA	DQA*01:02	DQA*03:01			DQA1*01:02:02	DQA1*03:03:01
B1988	HLA-DQB	DQB*03:01	DQB*05:02	DQB1*03:01	DQB1*05:02	DQB1*03:01:01	DQB1*05:02:01
B1988	HLA-DRB1	DRB1*04:01	DRB1*16:01	DRB1*04:01	DRB1*16:01	DRB1*04:01:01	DRB1*16:01:01

REFERENCES

1. Horton,R., Wilming,L., Rand,V., Lovering,R.C., Bruford,E.A., Khodiyar,V.K., Lush,M.J., Povey,S., Talbot,C.C. Jr., Wright,M.W., Wain,H.M., Trowsdale,J., Ziegler,A., Beck,S. (2004) Gene map of the extended human MHC. Nature Reviews Genetics, **5**, 889-899.
2. Forster, M., Forster,P., Elsharawy,A., Hemmrich,G., Kreck,B., Wittig,M., Thomsen,I., Stade,B., Barann,M., Ellinghaus,D., Petersen,B.S., May,S., Melum,E., Schilhabel,M.B., Keller,A., Schreiber,S., Rosenstiel,P., Franke,A. (2013) From next-generation sequencing alignments to accurate comparison and validation of single-nucleotide variants: the pibase software. Nucleic Acids Res., **41**, e16
3. Jia,X., Han,B., Onengut-Gumuscu,S., Chen,W.M., Concannon,P.J., Rich,S.S., Raychaudhuri,S., de Bakker,P.I. (2013) Imputing amino acid polymorphisms in human leukocyte antigens. PLoS One., **8,** e64683
